# Heat shock proteins in male infertility: recent advances and clinical implications

**DOI:** 10.3389/fcell.2026.1898983

**Published:** 2026-08-03

**Authors:** Zhongnan Wu, Wanting Zhang, Ruqi Han, Huiyu Ping, Linhui Xia, Haoran Ding, Jinxiang Yuan, Xiaomei Wang, Qin Qin

**Affiliations:** 1 Department of Medical Technology, Basic Medical College, Shanxi Medical University, Taiyuan, China; 2 Department of Biochemistry and Molecular Biology, Academy of Medical Sciences, Shanxi Medical University, Taiyuan, China; 3 Department of Biochemistry and Molecular Biology, Basic Medical College, Shanxi Medical University, Taiyuan, China; 4 College of Clinical Medicine, Jining Medical University, Jining, China; 5 Lin He’s Academician Workstation of New Medicine and Clinical Translation, Jining Medical University, Jining, China; 6 College of Basic Medicine, Jining Medical University, Jining, China; 7 Reproductive Medical Department, Shanxi Provincial People’s Hospital, Taiyuan, China

**Keywords:** heat shock proteins, heat shock transcription factors, heat stress, male infertility, spermatogenesis

## Abstract

The decline in male sperm quality has become a global concern for male reproductive health. Research indicates that heat stress is an important external factor that contributes to male infertility, potentially promoting germ cell apoptosis, disrupting redox balance, and affecting proteostasis. Recently, a growing body of research has demonstrated an association between heat stress-related proteins and male infertility. Heat shock proteins (HSPs) and their upstream transcription factors, known as heat shock factors (HSFs), constitute the core molecular system through which cells respond to heat stress. The main heat-shock protein families, including HSPA1, HSPA2, HSP90, HSP60, and small HSPs Small Heat Shock Proteins, play pivotal regulatory roles in spermatogenesis. These protein families influences the balance between germ cell repair and cell death by regulating proteostasis, oxidative homeostasis, and cell survival. The dysregulation of this system may represent an important molecular basis for heat stress-induced male infertility. This review focuses on the pathophysiological process of heat stress-induced male infertility, summarizes the potential mechanisms by which heat stress and heat stress-related proteins contribute to male infertility, and proposes a dynamic model of HSP systemic responses under heat stress. The current progress in the diagnosis and treatment of heat stress-related male infertility is summarized to provide a theoretical basis for further elucidating its pathogenesis, improving diagnostic accuracy, and guiding the developing of targeted therapeutic strategies.

## 1 Introduction

Male infertility is a global public health concern. Approximately 15% of couples worldwide experience infertility, and 2.5%–12% of men of reproductive age are estimated to be infertile, with marked regional variations (Atmoko et al., 2024). In recent decades, declines in sperm quality and quantity have been reported, drawing increasing attention to the effects of environmental- and lifestyle-related factors (Levine et al., 2023). Of these, heat stress is regarded as an important contributor to impaired male reproductive function (Xiao et al., 2024).

The optimal temperature for spermatogenesis in the testes is approximately 35 °C, which is 2 °C–3 °C lower than the core body temperature (Jorban et al., 2024). Consequently, the testes are highly sensitive to changes in temperature. Persistent factors, including high-temperature exposure, prolonged sedentary behavior, varicocele, and cryptorchidism, can elevate local testicular temperature, disrupt the spermatogenic microenvironment, and thus impair male fertility. Heat stress directly damages spermatogenic cells and triggers pathological changes, including oxidative stress, disruption of the blood–testis barrier, and a proteostasis imbalance, further affecting spermatogenesis (Qin et al., 2026). Specifically, increased temperature may promote reactive oxygen species (ROS) production and mitochondrial dysfunction, disrupt Sertoli cell–germ cell interactions, and induce apoptosis of spermatogenic cells. Heat shock proteins (HSPs) are pivotal protective factors in the male reproductive system.

Members of this protein family, such as HSP70, HSP90, HSP60, and small HSPs (sHSPs), assist in protein folding, prevent protein aggregation, maintain proteostasis, and exert anti-apoptotic effects (Hu et al., 2022). However, excessive or persistent heat stress can exceed the reparative capacity of HSPs, resulting in persistent or irreversible reproductive injury. Heat shock factors (HSFs), especially HSF1, may trigger pro-apoptotic signaling under severe stress conditions, further aggravating germ cell loss (Izu et al., 2004). Consequently, heat shock-related proteins may play a dual role in the pathogenesis of male infertility; an appropriate heat shock response helps protect spermatogenesis, whereas excessive stress or impaired HSP function may promote disease progression (Rizzoto et al., 2020; Kumar et al., 2026).

Based on this perspective, this article integrates the role of the HSP system in male infertility under heat stress from the standpoint of dynamic responses. It emphasizes that mild or transient heat stress primarily activates protective proteostasis-maintaining responses, whereas sustained or severe heat stress may exceed the compensatory capacity of the molecular chaperone system, thus promoting oxidative stress, apoptosis, and impaired spermatogenesis.

Currently, clinical interventions for heat stress-induced male infertility have mainly focused on managing underlying or contributing factors, including varicocele repair, lifestyle modification, and appropriate pharmacological treatment (Jensen et al., 2017; Gao et al., 2022; Brannigan et al., 2024). However, these approaches have limited applicability, often yield unsatisfactory therapeutic outcomes, and are associated with a high risk of recurrence. Thus, the development of novel and effective therapeutic strategies is needed. In recent years, the functions of heat shock-related proteins in the response to heat stress have been increasingly elucidated, placing them at the forefront of current research. Moreover, molecularly targeted therapeutic strategies have shown preliminary efficacy in this field and represent a promising direction for future clinical management. Elucidating the specific roles of heat shock-related proteins, particularly HSP70, HSP90, HSP60, sHSPs, and HSF1, in spermatogenesis, and exploring their potential utility as diagnostic biomarkers and therapeutic targets may have substantial translational significance.

## 2 Heat stress and male infertility

### 2.1 Developmental process of heat stress

Heat stress refers to a series of nonspecific physiological responses that occur when the body is exposed to high temperatures that exceed its thermoregulatory capacity, such as elevated heart rate and enhanced oxidative metabolism, ultimately impairing normal physiological functions (Lim, 2020). In the male reproductive system, the optimal temperature for spermatogenesis in the testes is approximately 35 °C. Maintenance of this temperature depends on testicular heat-dissipation mechanisms and the counter-current heat exchange system of the pampiniform plexus, which together support normal spermatogenesis (Aldahhan et al., 2021; De Toni et al., 2022).

The progression of heat stress can be divided into the compensatory and decompensatory phases (Li et al., 2024). When the ambient temperature increases or local heat dissipation is impaired, scrotal blood flow and sweating may increase to help maintain an appropriate testicular temperature and preserve spermatogenesis (Samir et al., 2023). However, when heat exposure is persistent or recurrent, these compensatory mechanisms become insufficient, leading to elevated testicular temperatures and reversible spermatogenic cell damage (Shahat et al., 2020). If the testicular temperature fails to return to the normal range or rises markedly, irreversible reproductive damage may occur, ultimately leading to spermatogenic arrest and subsequent male infertility (Kaushik et al., 2018). Therefore, the extent of heat stress-induced damage depends on temperature intensity as well as the duration and frequency of heat exposure and individual thermal adaptation capacity (Guzman-Echavarria et al., 2023; Robinson et al., 2023). Notably, acute heat stress may cause only a transient, reversible decline in sperm quality, whereas chronic heat stress, such as prolonged sedentary behavior or varicocele, can lead to permanent impairment of spermatogenesis (Eisenberg et al., 2023).

Experimental evidence supports a time-dependent progression of heat-induced testicular injury. In rats exposed to 43 °C, spermatogenic cell damage is mainly apoptotic after 30–45 min, whereas longer exposure for 60–90 min leads to more severe injury dominated by necrotic changes, suggesting a shift from apoptosis to necrosis under sustained heat stress (Kaushik et al., 2018). In rhesus monkeys subjected to scrotal heating at 43 °C for 30 min/day for six consecutive days, seminiferous epithelial disruption and germ cell exfoliation appeared on day 8, germ cell loss peaked between days 15 and 30, and gradual recovery occurred from days 45–90 (Rao et al., 2017). In healthy men exposed to scrotal heating at 43 °C for 30 min per session over 10 sessions, sperm concentration reached its nadir at week 8 and gradually recovered over the following 16 weeks (Rao et al., 2015). Together, these findings indicate that heat-induced injury and subsequent recovery are clearly time-dependent; however, differences in experimental models and exposure protocols preclude the definition of a uniform duration threshold.

### 2.2 Characteristics of heat stress-induced injury and compensatory responses in the testicular spermatogenic microenvironment

#### 2.2.1 Characteristics of heat stress–induced testicular injury

Heat stress-induced damage to the male reproductive system is mainly characterized by cell type specificity, temperature- and time-dependent effects, and the distinction between reversible and irreversible injuries. In terms of cell-type specificity, germ cells, especially spermatogonia and primary spermatocytes, are more susceptible to heat stress than Sertoli and Leydig cells, whereas somatic cells within the testicular microenvironment possess relatively higher heat tolerance (Zhang et al., 2006; Setchell, 2018). This discrepancy may be associated with the heightened sensitivity of germ cells to oxidative damage and proteostasis perturbation during active proliferation, meiosis, and chromatin remodeling. Heat stress can preferentially induce germ-cell injury through ROS accumulation, DNA damage, mitochondrial dysfunction, and activation of caspase-dependent apoptotic pathways (Robinson et al., 2023). Notably, Sertoli cells exhibit prominent heat resistance (Chen et al., 2008). Regarding temperature thresholds and time dependence, an increase in testicular temperature to approximately 37 °C may be sufficient to trigger germ cell apoptosis, and the severity of injury at a given temperature is positively correlated with the duration of heat exposure (Hirano et al., 2022; Jorban et al., 2024). With respect to reversibility, mild or single episodes of heat stress usually cause only a transient decline in sperm quality, which can recover after one to two spermatogenic cycles (Rao et al., 2015). In contrast, sustained or recurrent heat stress may induce Sertoli cell dysfunction and dedifferentiation, disrupt the blood–testis barrier, and ultimately lead to persistent spermatogenic impairment or, in severe cases, irreversible infertility (Hu et al., 2021; Hu et al., 2023).

#### 2.2.2 Heat stress adaptation of the spermatogenic microenvironment

The spermatogenic microenvironment refers to the local cellular and structural niche formed by Sertoli cells, Leydig cells, germ cells, and the blood–testis barrier, which provides the essential conditions for spermatogenesis, including nutrient support, hormonal signaling, and immune privilege, thereby supporting normal sperm development (França et al., 2016). During the early phase of heat stress, Sertoli cells may initiate supportive adaptive responses by upregulating HSPs and modulating energy metabolism. In contrast, germ cells, owing to their higher thermosensitivity, are prone to subsequent apoptosis and developmental arrest (Cai et al., 2021). Under heat stress, the testes are not merely subject to passive injury but can also activate compensatory protective mechanisms via the spermatogenic microenvironment to sustain spermatogenic homeostasis. The main compensatory mechanisms include increased scrotal and testicular blood flow; enhanced heat exchange via the pampiniform plexus; activation of the molecular chaperone system with upregulation of HSP70, HSP90, HSP60, and sHSPs; activation of antioxidant defense systems; and induction of autophagy (Kastelic et al., 2018; Shakeel et al., 2023; Hu Y. et al., 2024; Khan et al., 2024). When exposure to heat stress is mild and short-lived, these mechanisms may help preserve spermatogenic homeostasis. However, when misfolded and damaged proteins exceed the repair capacity of HSPs and ROS surpass the testicular antioxidant capacity, germ cells may exhibit irreversible injury and apoptosis, thereby contributing to male infertility.

### 2.3 Major mechanisms of heat stress-induced male infertility

Prolonged exposure to high temperatures does not impair male fertility via a single pathway. Instead, heat stress simultaneously affects germ cells, the spermatogenic microenvironment, and the testicular immune microenvironment. The central mechanism underlying HSP function involves the maintenance of protein homeostasis. Disruption of proteostasis is often accompanied by an imbalance between oxidative stress and antioxidant defense, ultimately promoting germ cell apoptosis (Santiago et al., 2020).

**FIGURE 1:**
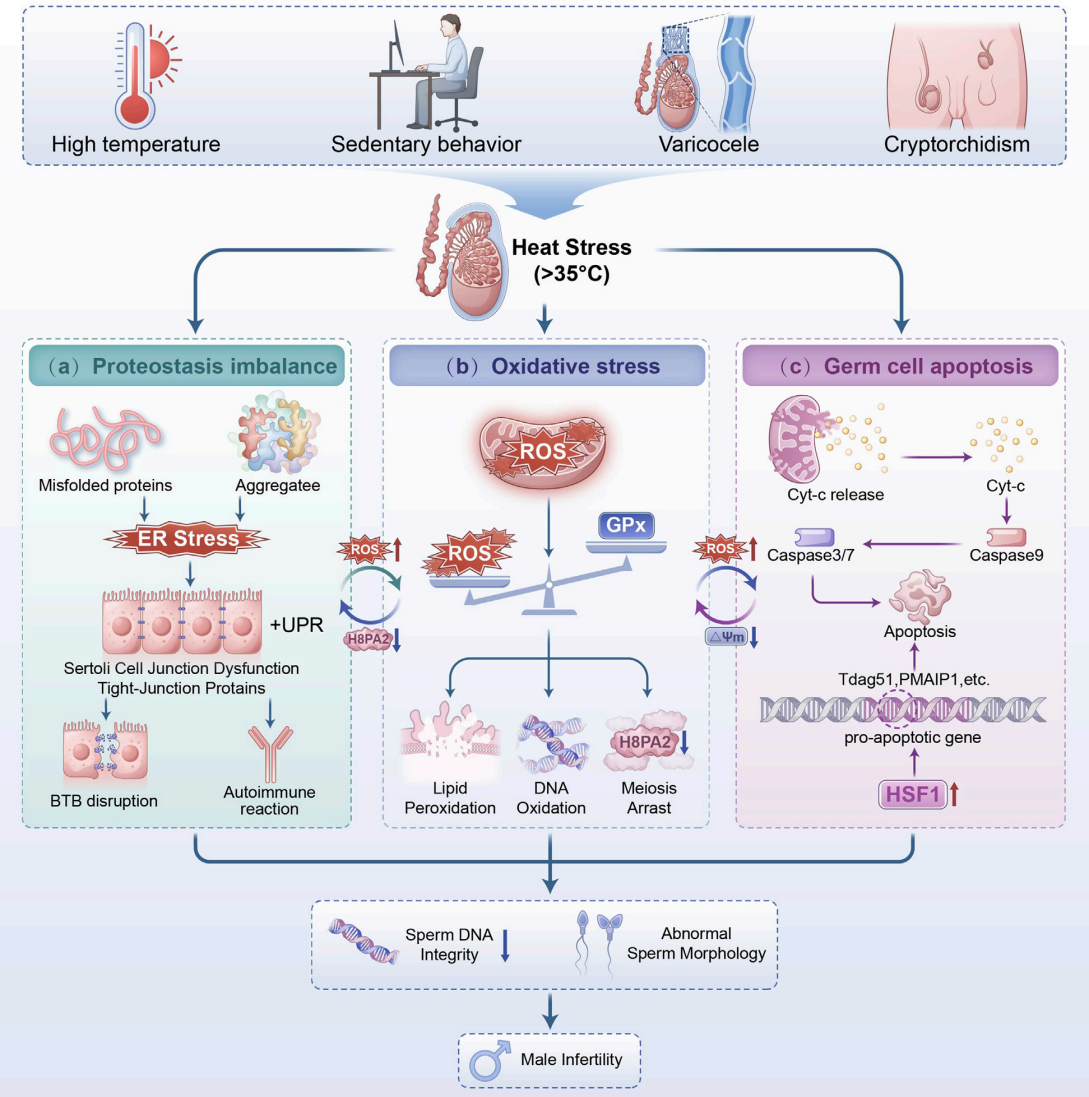
Proposed mechanisms by which testicular heat stress contributes to male infertility. Testicular heat stress induced by elevated temperatures, sedentary behavior, varicocele, or cryptorchidism disrupts proteostasis, increases oxidative stress, and triggers germ cell apoptosis. These alterations impair spermatogenesis through blood–testis barrier disruption, ROS-mediated molecular damage, meiotic arrest, and apoptotic loss of germ cells, ultimately leading to male infertility. **(a)** Proteostasis imbalance. **(b)** Oxidative stress. **(c)** Germ cell apoptosis.

High temperatures disrupt hydrogen bonds and hydrophobic interactions within protein structures, leading to misfolding, aggregation, and even endoplasmic reticulum stress, thereby initiating downstream cellular responses (Figure 1a) (Colon et al., 2017). Under normal conditions, misfolded proteins are recognized and bound by molecular chaperones, which assist in restoring their correct conformation. Alternatively, these proteins can be degraded via the ubiquitin-proteasome system or autophagy, thereby maintaining protein homeostasis (Margulis et al., 2020). However, under heat stress, the production of misfolded proteins may exceed the capacity of cellular protein quality-control systems. This leads to the accumulation of misfolded or aggregated proteins, the activation of endoplasmic reticulum stress and unfolded protein response, and further aggravation of heat-induced cellular injury (Hetz et al., 2020; Qin et al., 2021). In the testes, disruption of protein homeostasis can interfere with protein synthesis and folding during spermatogenesis. This may impair germ cell development and sperm function, induce Sertoli cell dysfunction, compromise the integrity of the blood–testis barrier, promote immune-related injury, and ultimately contributing to male infertility (Hu et al., 2023; Sugawara et al., 2025). Notably, disruption of protein homeostasis is not only a direct consequence of heat stress but also an important trigger for the activation of the HSP system (Alagar Boopathy et al., 2022). Exposed hydrophobic regions of misfolded proteins can recruit HSP70 and HSP90, thereby releasing HSF1 from inhibitory chaperone complexes and initiating the heat shock response (Rutledge et al., 2022; Ciccarelli and Andréasson, 2024).

Heat stress can also induce excessive accumulation of ROS in testicular tissues, thereby disturbing redox homeostasis (Figure 1b). Under physiological conditions, low concentrations of ROS play a crucial role in sperm capacitation, the acrosome reaction, and fertilization (Wagner et al., 2018; Aitken and Drevet, 2020). However, oxidative stress occurs when heat-induced ROS production exceeds the capacity of antioxidant defense systems, including superoxide dismutase, glutathione peroxidase, and catalase (He et al., 2022; Kowalczyk, 2022). HSPs are involved in this process. Oxidative stress can promote 4-hydroxy-2-nonenal (4-HNE)-mediated modification of HSPA2, targeting it for degradation through the ubiquitin-proteasome system, which may lead to the loss of proteins required for meiosis and contribute to spermatogenic arrest (Bromfield et al., 2017). In contrast, HSP60, HSP70, and HSP90 may help limit oxidative injury by maintaining protein homeostasis, stabilizing stress-sensitive proteins, and supporting cellular antioxidant responses (Hu et al., 2022). When ROS production exceeds the protective capacity of HSPs, oxidative stress may further aggravate proteostatic disruption and apoptosis, thereby amplifying heat stress-induced reproductive damage. Consequently, oxidative stress is not only a key amplifier of heat-induced injury but also an important mechanism contributing to male infertility.

Furthermore, multiple studies have demonstrated that heat stress-induced spermatogenic dysfunction 209 is closely associated with germ cell apoptosis, which displays both cell-type- and stage-specific 210 characteristics (Figure 1c) (Lue et al., 1999; Yoon et al., 2020). In particular, spermatogonia and primary spermatocytes are highly sensitive to heat stress and prone to apoptosis following heat exposure (Hirano et al., 2022; Chu et al., 2024). Germ cells in stages VII–VIII of the seminiferous epithelial cycle exhibit the most severe apoptotic response following heat exposure (Yoon et al., 2020). Heat stress induces germ cell apoptosis primarily via two pathways: the mitochondrial apoptotic pathway and the HSF1-mediated pro-apoptotic pathway (Janus et al., 2020). High temperatures increase mitochondrial membrane permeability and promote the release of cytochrome C, which in turn activates caspase-9 and the downstream caspase-3/7 cascade (Zou et al., 1999; Shakeri et al., 2017; Meng et al., 2026). This process leads to DNA fragmentation and ultimately germ cell death (Kaushik et al., 2019). The pro-apoptotic effect of HSF1is mediated by the induction of pro-apoptotic genes (Zhang et al., 2021).

More specifically, heat stress may first trigger proteostasis disruption, mitochondrial dysfunction, and ROS accumulation, followed by aggravated DNA damage, disruption of the blood–testis barrier, and activation of apoptotic responses. These events ultimately result in germ-cell loss, impaired spermatogenesis, and decreased semen quality. These mechanisms may occur independently or may amplify each other in a cascade, collectively forming a pathological network through which high temperatures contribute to male infertility.

## 3 Roles and response mechanisms of heat shock–related proteins in the pathogenesis of male infertility

### 3.1 Composition and functional basis of the HSF/HSP system in dynamic stress responses

Under heat stress, the testicular cells activate a series of protective responses, among which heat shock factors (HSFs) and heat shock proteins (HSPs) play central roles (Widlak and Vydra, 2017). These proteins collectively form a tightly regulated protective network that enables cells to maintain proteostasis and resist oxidative injury and apoptosis.

HSFs were first identified in *Drosophila melanogaster* (Ritossa, 1962). HSFs have since been confirmed to play essential roles in the initiation and regulation of heat shock responses (Rabindran et al., 1991). In mammals, HSFs are classified into various types, including HSF1, HSF2, HSF3, HSF4, HSF5, and HSFX/Y (Hästbacka et al., 2025). However, not all of these factors are directly associated with male fertility. Among fertility-related HSF members, HSF1 acts as the primary stress-responsive transcription factor. HSF1 is activated under heat stress to drive the expression of most HSPs, and participates in the regulation of sperm morphology and chromatin organization (Widlak and Vydra, 2017). HSF2 is primarily involved in organismal developmental differentiation and acts in concert with HSF1 during spermatogenesis (Singh et al., 2024b). HSF5 is specifically expressed in male germ cells and is mainly involved in meiosis under non-stress conditions rather than in the classical heat shock response (Liu et al., 2024; Yoshimura et al., 2024). Wang et al. reported that a single knockout of Hsf2 resulted in reduced testicular volume and mild spermatogenic abnormalities, whereas mice with a double knockout of Hsf1 and Hsf2 were completely infertile (Wang et al., 2004). These findings highlight the cooperative and essential roles of HSFs in spermatogenesis.

Furthermore, under heat stress, the expression of several HSPs is induced. These mainly include HSP70, which plays a central role in the refolding and clearance of misfolded proteins; HSP90, which participates in signal transduction and client protein maturation; HSP60, which assists in the correct folding of mitochondrial proteins; and HSP27, a member of the small HSP family, which protects cells by stabilizing the cytoskeleton and preventing protein aggregation (Hu et al., 2022). These factors play essential roles in heat stress response and are closely related to male reproductive function.

The HSP70 family is one of the most critical molecular chaperone families in heat stress repair; its function depends on the ATP-driven chaperone cycle and requires cooperation with HSP40 co-chaperones and nucleotide exchange factors (NEFs), which regulate substrate binding and release, promote correct protein folding, and prevent protein aggregation (Jiang et al., 2019). Based on their subcellular localization, HSP70 family members can be divided into cytosolic/nuclear HSP70s, including HSPA1, HSPA1L, and HSPA2; endoplasmic reticulum HSP70, represented by HSPA5; and mitochondrial HSP70, represented by HSPA9. Among these, HSPA1 is a strongly heat-inducible member that is highly expressed following heat stress (Maugeri et al., 2010). HSPA2 is a testis-enriched HSP70 member that is mainly distributed in spermatocytes and spermatids, where it participates in spermatogenesis and contributes to the quality control of spermatid proteins. HSPA5, also known as GRP78/BiP, functions as a major endoplasmic reticulum chaperone and regulator of endoplasmic reticulum stress responses (Wen et al., 2023). HSPA9, also known as mortalin, is primarily responsible for the maintenance of mitochondrial proteostasis (Esfahanian et al., 2023). Notably, HSPA9 is non-heat inducible, and its basal expression may be important for sustaining mitochondrial homeostasis during heat exposure (Kaul et al., 2002; Yoon et al., 2022).

The HSP90 family is mainly involved in the maturation, stabilization, and functional regulation of target proteins, rather than in the early folding of nascent polypeptides (Schopf et al., 2017; Morán Luengo et al., 2019). Under heat stress, HSP90 functions as a molecular chaperone that contributes to protein homeostasis and limits the aggregation of misfolded proteins. In addition, HSP90 is involved in the regulation of androgen receptor (AR) signaling and autophagy-related pathways, thereby participating in cellular stress adaptation (Rutledge et al., 2022; Wang et al., 2022; Shi et al., 2024). Key members of this family include HSP90α, HSP90β, GRP94, and TRAP1, of which HSP90α is an inducible protein and a key responder to heat-induced reproductive injury, closely associated with male infertility (Wei et al., 2024). Grad et al. demonstrated that HSP90α-knockout mice showed normal somatic development but exhibited meiotic arrest and male infertility (Grad et al., 2010).

The HSP60 family is primarily involved in the assembly of proteins in the mitochondrial matrix and the maintenance of protein homeostasis under stress. It also participates in the regulation of apoptosis pathways, with HSPD1 as a representative member that is localized in the mitochondrial matrix and functions as a molecular chaperone in an ATP-dependent manner (Singh et al., 2024a). In the mitochondria, HSP60 cooperates with HSP10 to form a chaperonin complex, thereby supporting mitochondrial protein folding, limiting excessive mitochondrial ROS production under heat stress, and maintaining the energy supply essential for sperm function (Malik and Lone, 2021; Hu et al., 2022). Animal studies have indicated that HSPD1 expression positively correlates with sperm motility parameters (Pang et al., 2022), suggesting that it may serve as a potential biomarker for male fertility.

sHSPs are ATP-independent, low-molecular-weight chaperones. After heat stress, their expression is induced via the classical HSF1-mediated pathway. In addition, some sHSPs, such as HSPB1/HSP27 and HSPB5, can be phosphorylated through kinase pathways, including the p38/MK2 pathway, which may regulate their chaperone activity, cytoskeletal interactions, and involvement in autophagy-related clearance of damaged cellular components (Gurgis et al., 2014; Gaestel, 2024; Cui et al., 2025). sHSPs preferentially bind to the exposed hydrophobic regions of misfolded proteins, thereby preventing irreversible aggregation, maintaining substrates in a refolding-competent state, and allowing them to be transferred to the HSP70 system for refolding or degradation (Ungelenk et al., 2016; Gonçalves et al., 2021; Reinle et al., 2022). Furthermore, sHSPs help to stabilize the cytoskeleton and membrane structure (Muranova et al., 2022; Gu et al., 2023). In the testis, HSP27, also known as HSPB1, is highly expressed in Sertoli cells, spermatogonia, and interstitial cells, where it stabilizes the cytoskeleton, acts in concert with HSP70 to counteract oxidative stress, and inhibits apoptosis (Adly et al., 2008; Muranova et al., 2022). HSPB9 and HSPB10 are predominantly expressed in the testes. HSPB9 is involved in spermatogenesis-related processes, whereas HSPB10 is essential for sperm head-tail coupling and normal sperm structure (de Wit et al., 2004; Hoyer-Fender, 2022). Xun et al. found that heat stress significantly upregulated the mRNA expression of HSPB9 and HSPB10 in a goat model (Xun et al., 2015). The loss or dysfunction of these proteins has been associated with impaired sperm differentiation and defects in head-tail coupling (Tedesco et al., 2022). However, relevant clinical evidence in humans remains limited.

In the protective response of the testis to heat stress, HSF1 acts as an upstream transcriptional regulator, whereas members of the HSP70 family, particularly HSPA1 and HSPA2, serve as key protective factors in maintaining germ-cell proteostasis and preventing apoptosis. HSP90, HSP60, and HSP27 are also involved in protein folding, mitochondrial homeostasis, and the regulation of cell survival (Purandhar et al., 2014; Scieglinska and Krawczyk, 2015). Taken together, these HSF/HSP members differ in both reproductive relevance and evidence strength, with some supported by established spermatogenic roles and others remaining preliminary biomarker or therapeutic candidates (Table 1).

**TABLE 1:** Major heat shock protein families involved in heat stress-induced male infertility.

Heat shock protein family	Key members	Subcellular localization	Core functions under heat stress	References
HSP70	HSPA1	Cytoplasm and nucleus	Anti-apoptotic, repairing damaged proteins and providing cytoprotection	Beere et al. (2000), Maugeri et al. (2010)
HSP70	HSPA2	Cytoplasm and nucleus	Repair damaged proteins, ensure sperm nuclear maturation and DNA integrity	Nowicka-Bauer et al. (2022)
HSP70	HSPA5/GRP78/BiP	Endoplasmic reticulum	Maintains endoplasmic reticulum protein homeostasis	Wang et al. (2017)
HSP70	HSPA9	Mitochondrial matrix	Non-heat-inducible, maintains mitochondrial homeostasis and participates in thermal injury protection	Kaul et al. (2002), Yoon et al. (2022)
HSP90	HSP90α/β, GRP94, TRAP1	Cytoplasm, endoplasmic reticulum, mitochondria	Assist protein folding and regulate androgen receptor signaling	Schopf et al. (2017)
HSP60	HSPD1	Mitochondrial matrix	Synergizes with HSP10, maintains mitochondrial protein homeostasis, and exerts anti-apoptotic effects	Malik and Lone (2021)
sHSPs	HSP27/HSPB1	Cytoplasm and others	Anti-apoptosis, anti-oxidation, cytoskeleton stabilization	Zou et al. (2023)

HSP70, heat shock protein 70; HSPA1, heat shock protein family A member 1; HSPA2, heat shock protein family A member 2; HSPA5, heat shock protein family A member 5; GRP78, glucose-regulated protein 78; BiP, binding immunoglobulin protein; HSPA9, heat shock protein family A member 9; HSP90, heat shock protein 90; GRP94, glucose-regulated protein 94; TRAP1, TNF, receptor-associated protein 1; HSP60, heat shock protein 60; HSPD1, heat shock protein family D member 1; HSP10, heat shock protein 10; sHSPs, small heat shock proteins; HSP27, heat shock protein 27; HSPB1, heat shock protein family B member 1.

### 3.2 Dynamic response phases of heat shock-related proteins under heat stress

Under heat stress, the protein response in testicular cells is a dynamic process. Based on the current evidence, this process can be conceptually divided into four interconnected phases: damage sensing, HSF activation and nuclear translocation, HSF transcription and translation with subsequent molecular chaperone activity, and finally repair or cell death (Kmiecik and Mayer, 2022; Zhang et al., 2022; Ciccarelli and Andréasson, 2024; Mailin et al., 2024).

### 3.3 Protein damage and the stress sensing

**FIGURE 2:**
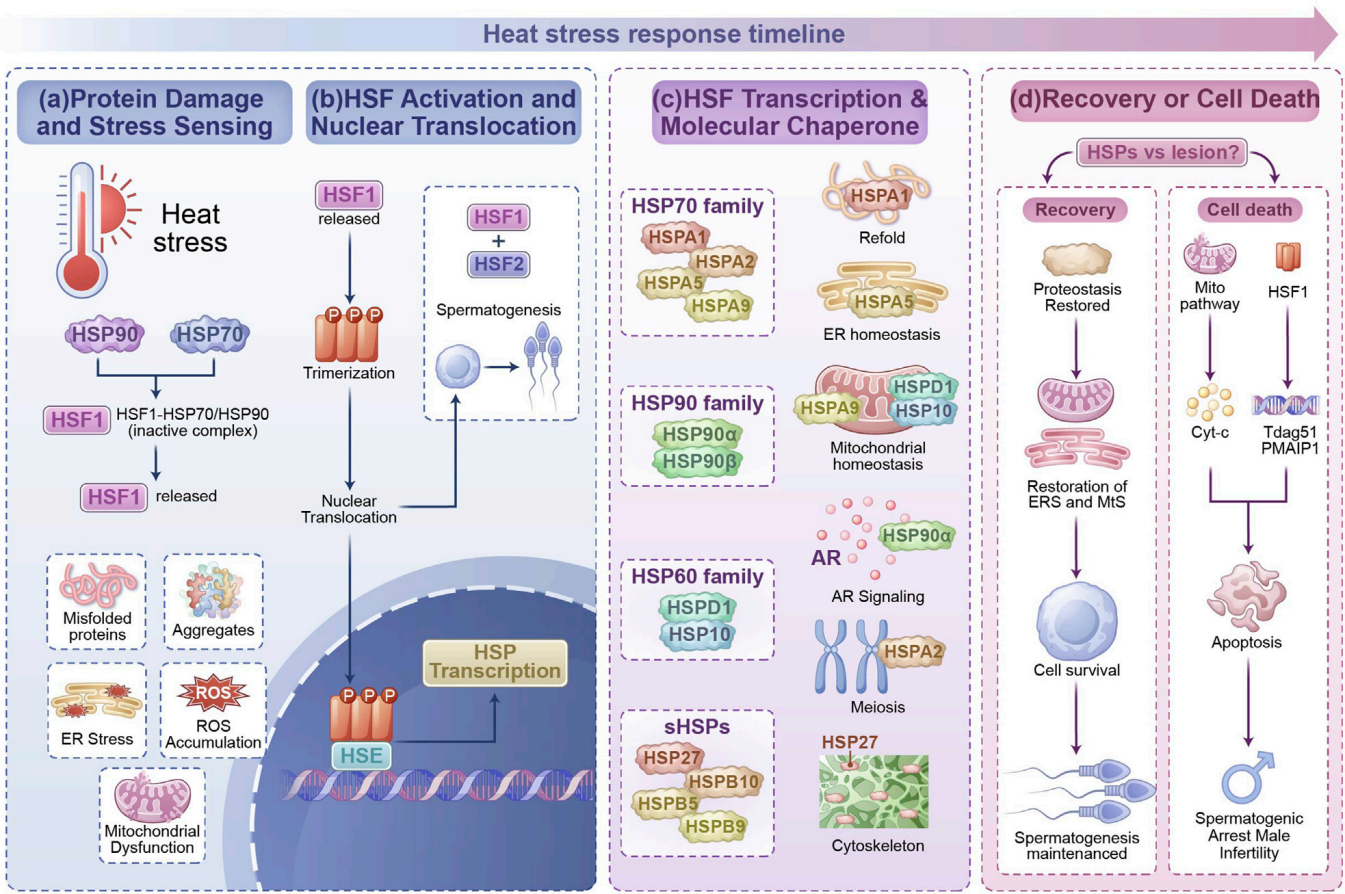
Proposed role of heat shock proteins in the regulation of testicular responses to heat stress. Elevated temperature resulting from environmental heat exposure, local testicular warming, varicocele, or cryptorchidism disrupts proteostasis and induces the accumulation of misfolded proteins. Activation of heat shock factor 1 (HSF1) promotes the expression of multiple heat shock proteins, including HSP70, HSP90, HSP60, and small HSP family members, which contribute to protein quality control, antioxidant protection, and cell survival. Under moderate heat stress, these adaptive responses preserve spermatogenesis and maintain testicular function. In contrast, severe or persistent heat stress causes mitochondrial dysfunction and activation of apoptotic signaling, leading to germ cell apoptosis, defective spermatogenesis, impaired sperm quality, and ultimately male infertility. **(a)** Protein damage and stress sensing. **(b)** HSF activation and nuclear translocation. **(c)** HSF transcription & molecular chaperone. **(d)** Recovery or cell death.

Cells convert heat-induced protein damage into a regulated transcriptional signal through a competitive release mechanism in which the dissociation of HSF1 from inhibitory chaperone complexes serves as a central switch in the stress response (Figure 2a) (Zheng et al., 2016). Under resting conditions, HSF1 is maintained in a latent or transcriptionally repressed state through interactions with molecular chaperones, including HSP90 and HSP70 (Vilaboa et al., 2017; Ciccarelli and Andréasson, 2024). Upon heat stress, the accumulation of misfolded proteins can titrate available chaperones, especially HSP70, away from HSF1, thereby relieving chaperone-mediated repression and promoting HSF1 activation (Kmiecik et al., 2020). Therefore, it can be inferred that the extent of HSF1 release is closely related to the intracellular burden of misfolded proteins. More severe protein damage can lead to greater HSF1 release and a stronger subsequent heat shock response. Thus, HSF1 activation is a critical step in the conversion of protein damage into a transcriptional response. If this step is impaired, cells may fail to respond rapidly to heat stress, resulting in the accumulation of misfolded or aggregated proteins and the subsequent impairment of spermatogenesis.

HSP70 and HSP90 act as sensors and play crucial roles during the sensing phase. Both bind to misfolded proteins to stabilize their structure; they also release HSF1 to initiate the subsequent transcriptional response (Ciccarelli and Andréasson, 2024). In yeast cells, high temperatures cause the cell membrane to stretch, which in turn leads to phosphorylation of the Hsp70 T492 site, forming a pathway that activates the heat shock response more rapidly than the traditional mechanism involving toxic proteins. This finding provides an important mechanistic framework for understanding the rapid activation of Hsf1 (Omkar et al., 2025). However, as the available evidence is primarily derived from yeast models, whether a comparable rapid heat-sensing mechanism operates in mammalian testicular cells or male germ cells remains to be determined. If the molecular chaperone functions of HSP70 and HSP90 are impaired by genetic deletion, mutation, or pharmacological inhibition, cells may become less efficient at recognizing, refolding, or clearing misfolded proteins, thereby compromising proteostasis recovery (Rutledge et al., 2022; Wentink et al., 2026).

The roles of sHSPs and HSP60 during the heat stress-sensing phase are limited; they primarily function in subsequent stages. Under heat stress, sHSPs (such as HSPB1/HSP27) can be phosphorylated and activated via the p38/MAPK-MK2 signaling pathway (Gallagher et al., 2024), thereby initiating autophagy to clear damaged organelles via the lysosomal pathway (Cui et al., 2025). This activation occurs early during heat stress; however, the subsequent signaling cascade takes time to unfold. sHSPs form complexes with misfolded proteins and recruit HSP40 and HSP70 to the substrates to complete the repair process (Gonçalves et al., 2021). In contrast, HSP60 primarily functions in the mitochondrial matrix to facilitate protein folding and requires the assistance of ATP and HSP10 (Caruso Bavisotto et al., 2023). Therefore, although sHSPs and HSP60 are not primary sensors during the sensing phase, they exert crucial functions in subsequent protective mechanisms.

Thus, protein damage and stress sensing represent the initiating phase of the dynamic HSF/HSP response model, determining whether the downstream heat shock response can be promptly activated.

### 3.4 HSF activation and nuclear translocation phase

Upon heat stress, HSF1 monomers are released from the inhibitory chaperone complexes and rapidly form trimers (Figure 2b). These trimers acquire DNA-binding activity and accumulate in the nucleus, where they bind to heat shock elements (HSEs) in the promoter regions of target genes and initiate HSP transcription (Feng et al., 2021). Therefore, HSF1 trimerization is a key step in DNA binding, whereas the rate of nuclear accumulation and duration of nuclear retention help determine the magnitude of HSP expression, indicating that this response is a tightly regulated signaling cascade (Simoncik et al., 2024). Although HSF2 is not the primary initiator of heat stress, it acts in concert with HSF1 during spermatogenesis to ensure the integrity of basal HSP expression and stress-induced responses (Himanen et al., 2022). Single knockouts of either Hsf1 or Hsf2 impair fertility, whereas double knockouts result in complete infertility (Widlak and Vydra, 2017). These findings highlight the essential role of the HSF1/HSF2-mediated heat shock pathway in spermatogenesis.

After dissociation from HSF1, HSP70/HSP90 preferentially bind to misfolded or denatured proteins. Because HSF1-mediated upregulation of HSP70/HSP90 requires transcription and translation, an increase in newly synthesized HSP70/HSP90 occurs with a delay. Accordingly, during the early phase of heat stress, cells primarily depend on preexisting molecular chaperones to refold, stabilize, or degrade abnormal proteins (Murshid et al., 2018; Alagar Boopathy et al., 2022). This implies that during the initial stages of heat stress, cells mainly rely on pre-existing cytoplasmic HSP70/HSP90. If reserves are insufficient, cells accelerate entry into cell death pathways, highlighting the importance of basal HSP70/HSP90 reserves. HSF activation and nuclear translocation constitute a pivotal step in the conversion of heat stress signals from cellular damage sensing into protective transcriptional responses.

### 3.5 HSP transcription, translation, and the molecular chaperone activation phase

As the transcription and translation of HSP genes are activated under the guidance of HSF1, the cell enters the molecular chaperone activity phase (Figure 2c) (Akerfelt et al., 2010). During this phase, HSP70 family members with different subcellular localizations form a comprehensive stress-protection network. HSPA1 is one of the most strongly induced members of the HSP70 family under heat stress. It recognizes misfolded or damaged proteins and promotes ATP-dependent refolding; when refolding fails, it helps direct these proteins toward degradation pathways (Rosenzweig et al., 2019). If HSPA1 induction is impaired, cells may have a reduced ability to tolerate heat-induced proteotoxic stress, which can contribute to severe damage during spermatogenesis (Williams et al., 2019; Tian et al., 2021). HSPA2 plays a crucial role in the maintenance of male fertility. Studies by Son et al. and Feng et al. suggested that reduced HSPA2 expression is associated with male infertility (Son et al., 2000; Feng et al., 2001). Recent evidence suggests that HSPA2 may serve as a potential biomarker for assessing spermatogenic status and fertilization potential (Nowicka-Bauer et al., 2022). In animal studies, mice carrying Eif4g3 mutations that downregulate HSPA2 expression exhibit complete infertility (Sun et al., 2010). This evidence consistently demonstrates the importance of HSPA2 in meiosis. Tian et al. reported that HSPA2 expression levels may serve as a non-invasive predictive marker of ICSI fertilization rates in patients with azoospermia (Tian et al., 2014). This finding suggests the potential clinical value of HSPA2 in the diagnosis and outcome prediction of male infertility and provides a new reference for disease evaluation. HSPA5/GRP78 mainly resides in the endoplasmic reticulum lumen as a key molecular chaperone regulating protein folding and the unfolded protein response (UPR). HSPA9/mortalin mediates the import, folding, and quality control of mitochondrial proteins (Wang et al., 2017; Ferré et al., 2021). Therefore, dysfunction of HSPA5 or HSPA9 aggravates heat-induced germ cell injury, thereby impairing sperm motility and quality (Liu et al., 2022; Wen et al., 2023).

The HSP90 family is not primarily involved in the early folding of nascent polypeptides; its primary function is to assist in the repair of misfolded proteins and restore the function of signaling proteins (Morán Luengo et al., 2019). In a mouse model, Kajiwara et al. demonstrated that Hsp90α is indispensable for maintaining normal spermatogenesis in adult males. Conditional knockout of Hsp90α resulted in aberrant AR expression in spermatogonia, followed by extensive apoptosis of post-pachytene germ cells, complete arrest of spermatogenesis, and eventual testicular atrophy (Kajiwara et al., 2012). Consistently, subsequent studies have further supported the pivotal roles of Hsp90α and AR signaling in regulating spermatogenic homeostasis and preserving male fertility (Cooke and Walker, 2021; Tang et al., 2021).

The HSP60 family, which is mainly localized in the mitochondrial matrix, plays a role in maintaining protein homeostasis; insufficient or defective expression may impair mitochondrial protein folding, thereby reducing sperm motility (He et al., 2020; Hauffe et al., 2025). The sHSP family participates in the early response to cellular stress by rapidly binding to unfolded or misfolded proteins and maintaining them in a refolding-competent state, which facilitates their subsequent refolding or degradation (Miller and Reichow, 2025). Furthermore, sHSPs play an important role in cytoskeletal protection (Muranova et al., 2022). Muranova et al. showed that mutations in HspB1 reduce its thermal stability, making it more susceptible to denaturation and aggregation (Muranova et al., 2020). Therefore, impaired sHSP function under heat stress may contribute to cytoskeletal disorganization, abnormal sperm morphology, and male infertility. This phase underscores the cytoprotective role of the HSP system, which maintains protein homeostasis through molecular chaperone activity and inhibits cell death.

### 3.6 Repair or cell death

Under heat stress, the fate of germ cells is largely determined by the balance between cellular damage and the protective capacity of HSPs (Figure 2d). When heat-induced damage exceeds the repair capacity of HSPs, the cell may undergo apoptosis (Williams et al., 2019). When HSPs successfully restore protein homeostasis, the cell may enter the repair and survival pathways, and when cellular damage exceeds the compensatory threshold, cell death pathways are activated (Somero and Physiology, 2020). Following heat stress, the expression of HSP70, HSP90, and sHSPs is upregulated, which promotes the refolding of misfolded proteins, facilitates the clearance of irreversible protein aggregates, and helps maintain organelle function, thereby supporting the survival of heat-sensitive germ cells and recovery of spermatogenesis (Maroto et al., 2025). When these repair mechanisms are successful, misfolded proteins are cleared or refolded, mitochondrial membrane potential is restored, and endoplasmic reticulum stress is alleviated, allowing cells to gradually return to a normal physiological state. Therefore, the protective capacity of HSPs is an important determinant of spermatogenic recovery following heat stress. Strong HSP-mediated protection may favor reversible injury, whereas insufficient protection may contribute to the persistent or irreversible impairment of spermatogenic function. Furthermore, the anti-apoptotic effects of HSPs are important during recovery.

HSP70, HSP90, and sHSPs delay or suppress apoptosis by directly modulating key apoptotic signaling pathways, thereby facilitating cell survival and repair (Verma et al., 2021). HSP70 acts at the apoptosome level in the intrinsic apoptotic pathway; by binding to Apaf-1, HSP70 induces conformational changes that mask the procaspase-9-binding site on Apaf-1, thereby preventing procaspase-9 recruitment to the apoptosome and inhibiting downstream caspase activation (Beere et al., 2000). HSP70 can also act directly upstream of the apoptotic pathway, exerting an anti-apoptotic effect by indirectly regulating Bax through the inhibition of the JNK pathway (Stankiewicz et al., 2005). Hsp90α, a member of the Hsp90 family, inhibits apoptosis by stabilizing Akt, suppressing caspase activation, and regulating members of the Bcl-2 family (Liu and Qian, 2024). HSP27, a member of the sHSP family, activates the anti-apoptotic kinase Akt and inhibits Bax activity, thereby suppressing apoptosis (Zou et al., 2023; Yang et al., 2026).

However, the HSP-mediated protection may be insufficient under severe or prolonged heat stress. Once cellular damage exceeds the compensatory threshold, HSPs may become functionally exhausted, allowing mitochondrial apoptotic signaling to predominate and ultimately inducing apoptosis (Wang et al., 2024). Simultaneously, autophagy is activated to remove damaged proteins and organelles, thereby contributing to the cellular response to irreversible injury (Hu Y. et al., 2024; Lei et al., 2025).

Heat stress can trigger an HSF1-centred pro-apoptotic program, whereby Tdag51, a pro-apoptotic gene, is specifically induced in pachytene-stage male spermatocytes. HSF1 binds to HSEs in the Tdag51 promoter region to activate its transcription, leading to Fas upregulation and eventual germ cell apoptosis (Hayashida et al., 2006). Janus et al. also demonstrated that, under heat shock conditions, HSF1 can directly bind to PMAIP1 (a pro-apoptotic gene) to induce cell death in mouse spermatocytes (Janus et al., 2020).

### 3.7 Limitations and dual roles of the HSF/HSP system

Although the HSP system constitutes a major defense mechanism by which the testes respond to heat stress, it is not infallible. Under mild or transient heat stress, HSF1-mediated HSP induction can facilitate protein refolding, prevent protein aggregation, preserve mitochondrial function, and promote germ-cell survival. Its protective capacity is strictly limited by the intensity and duration of thermal injury (Durairajanayagam et al., 2015; Capela et al., 2022). Under severe or sustained heat stress, unfolded and misfolded proteins rapidly accumulate and exceed the compensatory capacity of the proteostasis network. A large burden of misfolded substrates sequesters molecular chaperones such as Hsp70, thereby reducing the available chaperone pool and ultimately leading to the collapse of protein homeostasis (Ciccarelli and Andréasson, 2024). Notably, marked interindividual variability exists in sensitivity to heat stress and in the capacity for thermal adaptation (Fennel et al., 2023; Muhamad et al., 2025). Evidence from existing animal and cellular models suggests that this variability may be partly attributable to genetic variants affecting the HSF1/HSP-mediated heat shock response pathway, as well as to individual differences in basal and inducible molecular chaperone levels (Gibson et al., 2016; Rong et al., 2019; Nava and Zuhl, 2020; Hu L. et al., 2024).

Moreover, this system exhibits pronounced cell-type specificity: meiotic germ cells are far less tolerant to heat than Sertoli cells and are more prone to apoptosis (Shahat et al., 2020). When heat stress exceeds a critical threshold, excessive ROS burden and mitochondrial damage may indirectly lead to the structural and functional inactivation of HSPs (Bromfield et al., 2015; Zhao et al., 2021). Consequently, the system shifts from a repair-oriented mode to a cell death mode, during which HSF1 may participate in pro-apoptotic transcriptional programs to actively eliminate irreversibly damaged germ cells (Gaglia et al., 2020).

## 4 Clinical diagnosis and treatment strategies for male infertility

### 4.1 Diagnostic strategies

No specific criteria are currently available to diagnose heat stress-induced male infertility. The clinical diagnosis is primarily based on a combination of medical history (such as prolonged sitting, occupational heat exposure, and varicocele) and semen analysis while ruling out other potential causes (Giulioni et al., 2023). Although semen analysis may reveal abnormalities in the sperm concentration, motility, and morphology (Gül et al., 2024), these changes cannot be directly attributed to heat stress. Consequently, heat stress-induced male infertility is currently considered a diagnosis of exclusion, and more precise diagnostic criteria are required. In clinical practice, this condition should be interpreted as a comprehensive clinical assessment based on a history of heat exposure, abnormal semen parameters, and the exclusion of other potential causes, rather than as a distinct diagnostic entity with specific laboratory markers (Tables 2, 3).

**TABLE 2:** Comparison of HSP expression patterns across male reproductive disorders.

Disease	Major HSP types involved and trends in expression	Key findings	References
Varicocele	HSPA2: decreased expression; HSP70/HSP90: stress-related alterations	HSPA2 downregulation: impaired spermatogenesis, reduced sperm concentration, and sperm dysfunction HSP70/HSP90 alterations: local hyperthermia, oxidative stress, and disrupted proteostasis	Lima et al. (2006), Chan et al. (2013)
Cryptorchidism	HSP70: altered expression HSP90: limited clinical evidence	Elevated testicular temperature: heat-shock response and reduced germ-cell survival Altered HSP expression: impaired spermatogenesis and germ-cell apoptosis	Légaré et al. (2004), Kowalska et al. (2025)
Azoospermia	HSPA2: absent or markedly reduced in seminal plasma HSP90: limited direct evidence	HSPA2 deficiency: altered spermatogenic status Potential noninvasive marker of severe spermatogenic impairment and assisted reproductive outcomes	Nowicka-Bauer et al. (2022)
Heat stress	HSP70/HSP90: stress-induced upregulation; HSP60/HSC70: increased in some models; HSPA2: modest or model-dependent changes	HSP70/HSP90 upregulation: enhanced protein folding and proteostasis Prolonged heat exposure: germ-cell injury, increased apoptosis, and impaired spermatogenesis	Pei et al. (2012), Durairajanayagam et al. (2015), Mehta et al. (2024)

HSP, heat shock protein; HSPA2, heat shock protein family A member 2; HSP70, heat shock protein 70; HSP90, heat shock protein 90; HSP60, heat shock protein 60; HSC70, heat shock cognate 70.

**TABLE 3:** Direct research methods for heat stress-induced male reproductive injury and their relevance to HSP research.

Experimental method	Outcome measures	Association with HSPs	References
Heat-stress model induction combined with testicular histopathology	Seminiferous tubule injury; germ-cell sloughing; spermatogenic arrest	Assessment of heat stress–induced HSP expression Association between HSP alterations and testicular structural damage	Ribeiro et al. (2025)
Semen analysis	Sperm concentration; motility; morphology; kinematic parameters	Association between HSP alterations and impaired semen quality	Rao et al. (2016), World Health Organization (2021)
Oxidative stress and mitochondrial function assays	ROS; lipid peroxidation; antioxidant enzyme activity; mitochondrial membrane potential; ATP production; mitochondrial ROS	HSP-mediated protection against oxidative and mitochondrial damage Mechanistic link to heat-induced sperm dysfunction	Gong et al. (2017), Finelli et al. (2022)
DNA damage, chromatin integrity, and apoptosis assays	Sperm DNA fragmentation; abnormal chromatin condensation; germ-cell apoptosis; apoptotic pathway activation	HSP-mediated protection against heat-induced germ-cell injury	Agarwal et al. (2020)
Analysis of HSP expression	mRNA and protein expression levels of HSP70, HSPA2, HSP90, HSP60, HSP27, and related HSPs	Heat stress–induced activation or inhibition of specific HSP family members	Cinone et al. (2024)

HSP, heat shock protein; HSPs, heat shock proteins; HSP70, heat shock protein 70; HSPA2, heat shock protein family A member 2; HSP90, heat shock protein 90; HSP60, heat shock protein 60; HSP27, heat shock protein 27; ROS, reactive oxygen species; ATP, adenosine triphosphate; mRNA, messenger RNA; DNA, deoxyribonucleic acid.

Heat shock-associated proteins have shown considerable potential in this field. For instance, HSPA2 is upregulated in the testicular tissue of rats with varicocele (Afiyani et al., 2014), whereas clinical samples from patients with varicoceles show reduced HSPA2 expression (Panner Selvam and Agarwal, 2021). This discrepancy may be attributed to interspecies differences, disease progression, and a shift in the heat stress response from a compensatory to a decompensatory status. The available evidence is insufficient to support HSPA2 or other HSPs as established clinical diagnostic biomarkers for heat stress-related male infertility. HSPs still retain potential diagnostic significance and remain at the research stage as markers of reproductive damage. Standardization of testing samples and methods is still lacking, and marked individual and metabolic variations exist (Kaltsas et al., 2024). Therefore, the combined assessment of multiple HSPs and conventional semen parameters may hold important research value and represent a promising direction for future diagnostic developments. Further large-scale human studies are needed to validate the clinical utility of HSPs in risk assessment, evaluation of injury severity, and monitoring of therapeutic responses (Table 4).

**TABLE 4:** Evidence hierarchy and translational relevance of HSP/HSF-related candidate biomarkers and interventions in heat stress-associated male infertility.

HSP/HSF-related candidate biomarkers or interventions	Species/model system	Type of evidence	Key findings	Translational limitations	References
HSPA2 (candidate biomarker)	Human semen and testicular samples; ICSI studies; rat models	Human observational and animal evidence	Associated with sperm maturation, DNA integrity, and fertilization potential	Promising translational potential; no standardized cutoff values; limited specificity for heat stress	Feng et al. (2001), Tian et al. (2014), Nowicka-Bauer et al. (2022)
HSPA1/HSP70 (candidate mechanistic mediator)	Mouse and rat heat-stress models; cell-based models	Preclinical mechanistic evidence	Involved in protein repair, anti-apoptotic responses, and protection against heat stress	Primarily reflects a general stress response; lacks specificity for male infertility	Chen et al. (2008), Rosenzweig et al. (2019), Williams et al. (2019), Tian et al. (2021)
HSPA5 and HSPA9 (candidate mechanistic mediators)	Cell-based and animal models; limited human sperm studies	Preclinical evidence with limited human observational data	Maintenance of endoplasmic reticulum and mitochondrial homeostasis	Insufficient direct evidence in human heat stress–related male infertility	Wang et al. (2017), Liu et al. (2022), Wen et al. (2023)
HSF1/HSF2 (upstream regulators)	Genetically modified mouse models; cellular heat-stress models	Animal genetic and mechanistic evidence	Regulation of HSP expression and spermatogenesis	Complex biological roles; substantial risks associated with direct pharmacological targeting	Izu et al. (2004), Wang et al. (2004), Widlak and Vydra (2017), Janus et al. (2020)
HSF5 (spermatogenesis-associated factor)	Human genetic studies; mouse models	Human genetic evidence with animal validation	Associated with spermatogenic arrest during meiotic prophase	Indirect association with heat stress	Liu et al. (2024), Yoshimura et al. (2024)
HSP90 family (mechanistic mediators/potential therapeutic targets)	Knockout mouse models; cell-based models	Animal genetic and mechanistic evidence	Protein homeostasis; androgen receptor signaling; spermatogenesis	Broad biological functions; caution required for clinical targeting	Grad et al. (2010), Kajiwara et al. (2012), Schopf et al. (2017), Tang et al. (2021)
HSP60/HSPD1 (mitochondria-associated molecule)	Sperm studies in mice, boars, and other animal species	Animal and mechanistic evidence	Mitochondrial protein folding; sperm motility	Limited direct extrapolation from animal studies to humans	Chen et al. (2008), Purandhar et al. (2014), Pang et al. (2022)
HSP27/HSPB1, HSPB9, and HSPB10 (small HSPs/candidate mechanistic mediators)	Caprine heat-stress models; other animal and cell-based models	Preclinical evidence	Antioxidant and anti-apoptotic activity; cytoskeletal stabilization	Marked species- and tissue-specificity; lack of human validation	de Wit et al. (2004), Adly et al. (2008), Purandhar et al. (2014), Xun et al. (2015)
CoQ10, resveratrol, vitamin C, melatonin, etc. (non-specific adjunctive interventions)	Human studies of male infertility; animal models	Human clinical and preclinical evidence	Antioxidant and anti-apoptotic effects; improvements in selected semen parameters	Not specific to HSP/HSF pathways; substantial study heterogeneity	Bae et al. (2014), Shadmehr et al. (2018), Sun et al. (2019), Zhang et al. (2020), Zhao et al. (2021)
GGA, Ashitaba, L-theanine/DHM, baicalin, etc. (experimental HSP/HSF-modulating interventions)	Chinese sturgeon sperm-preservation models; mouse models; cell-based models	Species-specific and preclinical evidence	Potential attenuation of heat stress–induced damage through modulation of the HSF/HSP axis	No direct human clinical evidence; safety evaluation and human trials required	Xi et al. (2018), Kokubu et al. (2019), Sui et al. (2019), Yang et al. (2024)
Baicalin-related molecular docking with HSP70/HSP90 and other HSP-related targets	In silico models	Computational evidence	Predicted binding to potential HSP targets	Hypothesis-generating only; does not confirm physical binding or therapeutic efficacy	Sui et al. (2019), Kumari et al. (2026)

Evidence was stratified according to species/model system, type of evidence, and proximity to human clinical application. Human observational or clinical evidence was considered more directly translatable, whereas animal, cell-based, species-specific sperm preservation, and *in silico* studies were regarded as preclinical or hypothesis-generating evidence. CoQ10, coenzyme Q10; DHM, dihydromyricetin; GGA, geranylgeranylacetone; ICSI, intracytoplasmic sperm injection.

### 4.2 Treatment strategies

**FIGURE 3:**
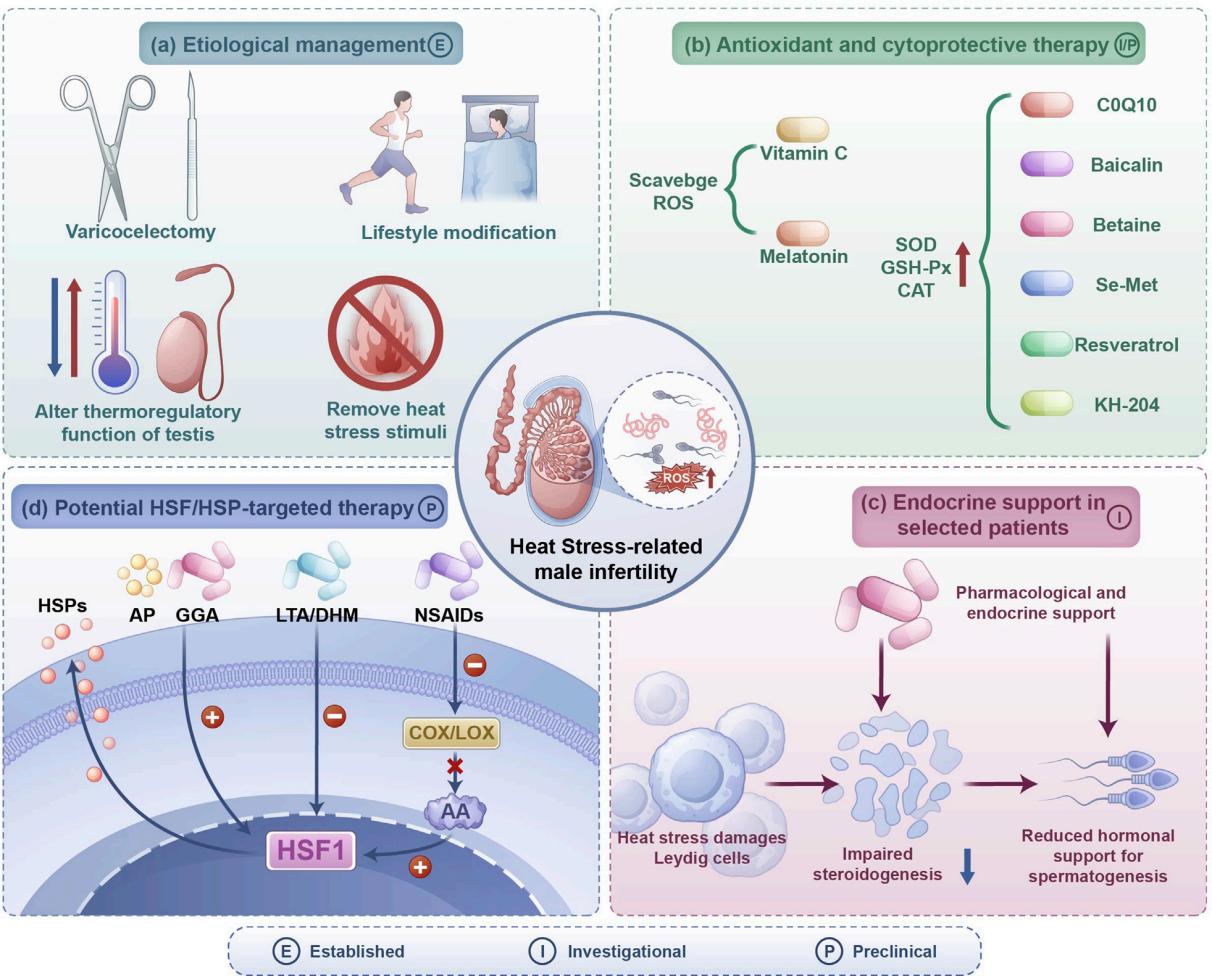
Therapeutic strategies for heat stress-related male infertility according to their stage of development. **(a)** Etiological management, including varicocelectomy, restoration of testicular thermoregulation, lifestyle modification, and avoidance of heat exposure, represents established clinical practice. **(b)** Antioxidant and cytoprotective therapies are currently being explored in clinical and preclinical settings, whereas HSF/HSP-targeted interventions remain mainly preclinical. **(c)** Endocrine support may be considered in selected patients but remains an investigational, patient-specific strategy. **(d)** Potential HSF/HSP-targeted therapy. Abbreviations: ROS, reactive oxygen species; HSF, heat shock factor; HSP, heat shock protein; SOD, superoxide dismutase; GSH-Px, glutathione peroxidase; CAT, catalase; CoQ10, coenzyme Q10; NSAIDs, nonsteroidal anti-inflammatory drugs.

Current treatments for heat stress-induced male infertility include etiological management, lifestyle modifications, and pharmacological interventions (Shahat et al., 2020). According to the current level of evidence, these therapeutic strategies can be further categorized into established clinical interventions, adjunctive therapies with inconsistent evidence, and experimental HSP/HSF-targeted approaches. Basic treatments include varicocelectomy and lifestyle interventions aimed at reducing scrotal heat exposure (Figure 3a) (Gao et al., 2022). Among these, avoiding prolonged heat exposure, reducing sauna or hot bath use, minimizing prolonged sitting, improving occupational protection, and managing clinically relevant varicocele are relatively well-established and feasible interventions. Although these measures partially improve testicular thermoregulation, their efficacy is limited when irreversible testicular damage occurs.

Pharmacological treatments primarily focus on antioxidant and cytoprotective strategies (Figure 3b). Coenzyme Q10 has been shown in multiple studies to improve sperm parameters (Akhigbe et al., 2025; Bakri et al., 2025). Resveratrol may protect testicular function by activating sirtuin 1 (SIRT1), thereby exerting antioxidant and anti-apoptotic effects (Zhang X. et al., 2024; Chimento et al., 2025). As a strong reducing agent, VC can directly neutralize ROS by donating electrons, thereby reducing oxidative stress levels (Sun et al., 2019). Melatonin also exhibits direct free radical scavenging activity (Zhang et al., 2020). Compounds in traditional Chinese medicines, including KH-204 (Bae et al., 2014), selenomethionine (Se-Met) (Xu et al., 2024; Zhang Z. et al., 2024), betaine (Cabezón et al., 2016; Shadmehr et al., 2018), and baicalin (Guo et al., 2015; Sui et al., 2019), can reduce testicular ROS levels and suppress apoptosis by upregulating antioxidant enzyme systems. It should be noted that the evidence supporting the aforementioned antioxidant and cytoprotective therapies remains heterogeneous, with considerable variation across studies in terms of study populations, dosage, treatment duration, and outcome measures. Therefore, these interventions cannot currently be regarded as standardized treatment regimens for heat stress-induced male infertility.

Heat stress may impair testicular endocrine cells and reduce androgen production. TTherefore, endocrine-based therapy may help maintain an appropriate hormonal environment for spermatogenesis in selected cases (Figure 3c) (Kathrins and Niederberger, 2016; Schlegel et al., 2021). Although these pharmacological and hormonal treatments may provide some therapeutic benefit, they mainly relieve symptoms and delay disease progression rather than provide a definitive cure. The HSPs discussed in this review may represent promising therapeutic targets for HS-induced male infertility. However, this direction remains largely at the experimental or translational research stage.

Several agents may directly or indirectly modulate the HSP/HSF1 axis (Figure 3d). For example, geranylgeranylacetone (GGA) has been reported to activate HSF1 and increase HSP90α expression in frozen sperm from male Chinese sturgeon, thereby helping preserve sperm quality (Xi et al., 2018). GGA may also induce the expression of other HSPs and exert a broader cytoprotective effect (van Marion et al., 2020). Nonsteroidal anti-inflammatory drugs (NSAIDs) target and inhibit cyclooxygenase (COX), thereby blocking the COX-mediated metabolism of arachidonic acid (AA); in certain contexts or with specific agents, they also inhibit lipoxygenase (LOX) (Meshram et al., 2021; Mukhopadhyay et al., 2023). As a potent inducer of HSF activation, AA upregulates HSF activity and subsequently induces HSP accumulation, ultimately enhancing cellular thermotolerance (Jurivich et al., 1994; Kokubu et al., 2019). In a mouse spermatocyte model, Artemisia annua powder (AP) enhanced HSP production by upregulating Hsf1 mRNA expression and maintaining HSF2 levels, thereby ensuring that key proteins for spermatogenesis are not lost; this treatment markedly attenuated heat-induced spermatocyte damage after exposure to a 41 °C water bath for 15 min (Kokubu et al., 2019). L-theanine (LTA) and dihydroquercetin (DHM), either alone or in combination, interfere with the transduction of heat stress injury signals via direct downregulation of HSF1, which further suppresses the synthesis of HSP27 and HSP70, thereby safeguarding reproductive function and sperm quality in mice (Yang et al., 2024). Furthermore, Hou et al. used proteomic approaches and identified HSP70 and HSP90 as potential targets mediating the protective effects of baicalin (Hou et al., 2022). Computational protein modeling studies further suggested that baicalin possesses a high predicted affinity for HSP70 and HSP27, indicating a possible direct interaction with these proteins and a potential role in regulating heat stress-related protective mechanisms (Kumari et al., 2026). These findings support the therapeutic potential of targeting HSPs and provide a new direction for treating patients with heat stress-induced male infertility.

It should be emphasized that the evidence supporting the role of the HSP-HSF1 axis in male infertility is mainly derived from animal models, cellular models, sperm cryopreservation studies, or computational prediction analyses, and sufficient human clinical evidence is still lacking. Future studies involving standardized animal experiments, prospective population-based studies, and clinical trials are required to further clarify their efficacy, safety, and clinical translational potential.

## 5 Summary and outlook

Heat stress-induced spermatogenic dysfunction in males is not caused by a single form of heat damage but rather results from the synergistic activity of multiple pathways, including protein homeostasis imbalance and oxidative stress accumulation, which collectively impair germ cells and disrupt spermatogenesis. Although heat stress can initially activate protective responses such as HSP induction and antioxidant defense, prolonged or excessive stress may overwhelm these protective mechanisms and shift the cellular response to injury and cell death. Therefore, HSPs are not only involved in maintaining cellular homeostasis under stress but also serve as key molecular links between heat stress and the pathogenesis of male infertility. Heat stress-related proteins may serve as markers for disease staging and guide clinical treatment decisions.

Overall, HSF1, HSF2, HSPA1, HSPA2, HSP90, and certain sHSPs are among the heat shock-related factors most closely associated with heat stress and male infertility in current research. Among these, HSF1 serves as a central activator of HSP expression under heat stress and promotes pro-apoptotic signaling in spermatocytes under severe stress conditions. HSPA2 has been proposed as a candidate biomarker for impaired spermatogenesis, given that its abnormal expression has been detected in cases of human male infertility. In addition, animal models with disrupted heat shock-related genes or pathways, including those associated with HSPA5 and HSP90, further suggest that aberrant HSP expression or dysfunction may contribute to meiotic arrest, spermatogenic failure, and even loss of fertility.

These findings provide an important basis for identifying molecular biomarkers and therapeutic targets of heat-induced male infertility. However, there are several limitations in this field. For instance, most empirical evidence is derived from animal models, such as mice and boars, whereas studies using human clinical samples remain limited. Inconsistencies in the heat exposure methods and durations across different experiments lead to potential biases in the results. However, the roles of various HSPs under heat stress have not yet been sufficiently explored.

Future studies should shift from mechanistic interpretation to experimental validation. Establishing standardized *in vivo* and *in vitro* heat-stress models with consistent temperature, exposure duration, and recovery time would improve the comparability of findings across studies. In combination with genetic approaches such as knockout or overexpression, these models may help clarify the expression patterns and functional roles of key HSPs/HSFs in spermatogenic cells, apoptosis, and blood–testis barrier integrity. Moreover, integrated assessment of ROS levels, oxidase activity, and epigenetic alterations, including DNA methylation and histone modifications, may further elucidate the links among oxidative stress, HSP dysregulation, and male infertility. Future studies should investigate the mechanisms by which heat stress impairs male fertility, expand the use of human clinical samples, and integrate proteomic and multiomics approaches. These efforts may help identify more precise molecular mechanisms and support the development of personalized strategies to restore or improve fertility.

## 6 Literature search strategy

This review searched PubMed and Google Scholar for English-language articles published up to June 2026. The search terms included “heat stress,” “testicular heat stress,” “male infertility,” “spermatogenesis,” “heat shock proteins,” “HSP70,” “HSP90,” “HSP60,” “small heat shock proteins,” “oxidative stress,” “apoptosis,” “diagnosis,” and “treatment,” which were used in various combinations according to the topic of interest. Priority was given to reviews and representative animal and cellular studies closely related to heat stress-associated proteins and male infertility. The reference lists of the included articles were also screened to identify additional relevant studies.
